# Hemodynamic parameters and diabetes mellitus in community-dwelling middle-aged adults and elders: a community-based study

**DOI:** 10.1038/s41598-024-62866-7

**Published:** 2024-05-27

**Authors:** Tzu-Wei Wu, Yih-Jer Wu, Chao-Liang Chou, Chun-Fang Cheng, Shu-Xin Lu, Li-Yu Wang

**Affiliations:** 1 Department of Medicine, MacKay Medical College, No. 46, Sec. 3, Jhong-Jheng Rd., San-Jhih District, New Taipei City, Taiwan; 2 Institute of Biomedical Sciences, MacKay Medical College, New Taipei City, Taiwan; 3 Cardiovascular Center, Department of Internal Medicine, MacKay Memorial Hospital, Taipei, Taiwan; 4 Department of Medical Research, MacKay Memorial Hospital, Taipei, Taiwan; 5 Department of Neurology, MacKay Memorial Hospital, New Taipei City, Taiwan; 6 Tamsui Health Station, Department of Health, New Taipei City Government, New Taipei City, Taiwan

**Keywords:** Carotid blood flow, Pulsatility index, Resistance index, Case-control study, Community-based, Diabetes mellitus, Hemodynamics, Prognostic markers, Diabetes

## Abstract

Hemodynamic parameters have been correlated with stroke, hypertension, and arterial stenosis. While only a few small studies have examined the link between hemodynamics and diabetes mellitus (DM). This case-control study enrolled 417 DM patients and 3475 non-DM controls from a community-based cohort. Peak systolic velocity (PSV), end-diastolic velocity (EDV), blood flow velocity (MFV), pulsatility index (PI), and the resistance index (RI) of the common carotid arteries were measured by color Doppler ultrasonography. Generalized linear regression analyses showed that as compared to the non-DM controls, the age-sex-adjusted means of PSV, EDV, and MFV were − 3.28 cm/sec, − 1.94 cm/sec, and − 2.38 cm/sec, respectively, lower and the age-sex-adjusted means of RI and PI were 0.013 and 0.0061, respectively, higher for the DM cases (all *p*-values < 0.0005). As compared to the lowest quartiles, the multivariable-adjusted ORs of DM for the highest quartiles of PSV, EDV, MFV, RI, and PI were 0.59 (95% confidence interval [CI] 0.41–0.83), 0.45 (95% CI 0.31–0.66), 0.53 (95% CI 0.37–0.77), 1.61 (95% CI 1.15–2.25), and 1.58 (95% CI 1.12–2.23), respectively. More importantly, the additions of EDV significantly improved the predictabilities of the regression models on DM. As compared to the model contained conventional CVD risk factors alone, the area under the receiver operating curve (AUROC) increased by 1.00% (95% CI 0.29–1.73%; *p *= 0.0059) and 0.80% (95% CI 0.15–1.46%; *p* = 0.017) for models that added EDV in continuous and quartile scales, respectively. Additionally, the additions of PSV and MFV also significantly improved the predictabilities of the regression models (all 0.01 < *p*-value < 0.05). This study reveals a significant correlation between DM and altered hemodynamic parameters. Understanding this relationship could help identify individuals at higher risk of DM and facilitate targeted preventive strategies to reduce cardiovascular complications in DM patients.

## Backgroud

Atherosclerosis is a chronic disease that causes the occlusion of arteries by the accumulation of plaques within the arterial intima^1^. These plaques consist of lipids, predominantly low-density lipoprotein (LDL), and inflammatory cells, such as macrophages that transform into foam cells after phagocytosing lipids^2,3^. Atherosclerosis advances gradually and often asymptomatically, but it can be aggravated by other factors such as hypertension^4^. As the plaques enlarge, they can impair blood flow and induce shear stress in the vessel wall. This can provoke the erosion of vulnerable plaques and the generation of thrombi that can occlude the artery or embolize other organs^5^. Atherosclerosis can result in severe cardiovascular complications such as myocardial infarction and stroke, which are among the leading causes of mortality worldwide^6,7^. Atherosclerosis is especially common in developed countries, but it is also increasing in developing countries^8^. In Taiwan, for instance, five of the top ten causes of mortality are associated with atherosclerosis^9^.

Hemodynamics is the study of blood flow and the forces acting on the blood vessels and the heart. The relationship between atherosclerosis and hemodynamics is complex and bidirectional. On one hand, hemodynamic shear stress can influence the development and progression of atherosclerosis by modulating the phenotype and function of endothelial cells and smooth muscle cells, and by promoting or inhibiting inflammation, oxidative stress, lipid accumulation, and matrix remodeling in the arterial wall^10–12^. On the other hand, atherosclerosis can alter the geometry and elasticity of the arteries, which can affect the hemodynamic patterns and parameters such as pressure, flow, velocity, and shear stress^13^. These changes can further influence the stability and rupture risk of atherosclerotic plaques. Key hemodynamic parameters include peak systolic velocity (PSV), end-diastolic velocity (EDV), and mean blood flow velocity (MFV) measured by Doppler ultrasonography. Pulsatility index (PI) and resistance index (RI) were secondary parameters calculated from velocities^14,15^ and were accepted as methods of examing microcirculation with a variety of clinical applications^16^. PI is defined as the difference between PSV and EDV, divided by MFV, and RI is defined as the difference between PSV and EDV, divided by PSV.

Diabetes mellitus (DM) is a metabolic disorder characterized by chronic hyperglycemia that induces polyuria, polydipsia, and polyphagia. DM results from inadequate insulin secretion and/or impaired insulin action in the target tissues^17^. There are two main types of diabetes: type 1 and type 2. Type 1 diabetes is an autoimmune disease that causes β-cell destruction in the pancreatic islets. It typically manifests in children and adolescents and necessitates exogenous insulin therapy. Type 2 diabetes is more prevalent and involves insulin resistance that exacerbates as the β-cell function deteriorates^18^. DM affects over 450 million people worldwide and accounts for 4.2 million deaths annually^19^. DM is diagnosed by assessing fasting and post-load plasma glucose levels.

Clinically, DM is associated with increased risks of vascular events, including carotid artery diseases^20,21^. Our previous study demonstrated the prevalence of DM is significantly associated with the development and severity of carotid atherosclerosis^22^. Later we identified 9 DM SNPs showing promising associations with the presence of carotid plaque in a community-based case-control study^23^. The associations of hemodynamics and carotid pulsatility with DM were noted in a few studies previously^24–26^. However, this clinical correlation is not fully explored. In this community-based case-control study, the relationship between DM and hemodynamic parameters was investigated in more than 3800 subjects, including 417 DM patients and 3475 non-DM controls, from the Northern coast of Taiwan.

## Methods

### Study subjects

The study subjects were recruited from our two previous community-based cohort studies that enrolled 40–74-year-old middle-aged adults and elders residing in the five districts in the northern coastal area of Taiwan for at least six months^22,27^. Cohort I and II enrolled study subjects from September 2010 to May 2011 and from September 2014 to May 2020, respectively. During each period, well-informed invitation letters describing the objective and protocols of the study were sent to households with eligible subject(s), and recruitment sites were set up at the local health stations, schools, or community activity centers. Residents who were willing to complete a structured questionnaire regarding personal health information and willing to provide blood samples were recruited. A total of 4102 residents voluntarily provided informed consent and were enrolled. Subjects who had a positive history of physician-diagnosed myocardial infarction or had ever received a cardiac catheter or stent (n = 165) and who were without a proper flow pattern sample (n = 45) were excluded, leaving a total of 3892 middle-aged adults and elders in this study. The study complied with the 1975 Helsinki Declaration on ethics in medical research and was reviewed and approved by the institutional review boards of MacKay Medical College (No. P990001) and MacKay Memorial Hospital (No. 14MMHIS075).

### Anthropometric and biochemical measurements

The measurements of baseline anthropometric and clinical characteristics were described previously^27,28^. In brief, blood pressure was measured three times by a digital system (UDEX-Twin; ELK Co., Daejon, Korea) in the morning after 10 min of rest. Three blood pressure measurements, with an interval of ≥ 3 minutes, were made for each participant. The averages of repeated measurements of systolic blood pressure (SBP) and diastolic blood pressure (DBP) were used for analyses.

A venous blood sample was collected from each participant for blood lipids and glucose analyses after at least 10 hours of fasting. We used an autoanalyzer (Toshiba TBA c16000; Toshiba Medical System, Holliston, MA, USA) to determine the blood levels of lipids, including total cholesterol (TCHO), high-density lipoprotein cholesterol (HDL-C), low-density lipoprotein cholesterol (LDL-C), and triglycerides (FTG), and glucose (FPG) with commercial kits (Denka Seiken, Tokyo, Japan).

In this study, DM was defined as FPG ≥ 126 mg/dL or the use of insulin or other hypoglycemic agents. Hypertension was defined as SBP ≥ 140 mmHg, DBP ≥ 90 mmHg, or a history of taking antihypertensive medications. Current cigarette smoking was defined as having smoked cigarettes at least 4 days per week during the past month before enrollment. Current alcohol drinking was defined as having drunk alcohol-containing beverages at least 4 days per week during the past month before enrollment.

### Ultrasonographic measurements of carotid blood flow

In the study, blood flows, including PSV, EDV, and MFV, of extracranial carotid arteries, were measured at the middle segment of the bilateral common carotid arteries by color Doppler ultrasonography. The ultrasonographic systems (GE Healthcare Logie E, Vivid 7, and Vivid E9; General Electric Company, Milwaukee, USA), which were equipped with a multi-frequency linear array transducer, were operated by two experienced technicians who were blind to patients’ clinical profiles. Each participant was examined in the supine position with his/her head turned 45° from the site being measured. An insonation angle equal to or less than 60° and a sample volume size covering 1/2–2/3 of the arterial lumen were maintained for all Doppler measurements. In the study, a proper flow pattern sample was defined as subjects with at least 3 waveforms with similar patterns. The subject’s PI and RI were calculated as (PSV-EDV)/MFV and (PSV-EDV)/PSV, respectively. In the study, the averages of the measurements of the right and left common carotid arteries were used for statistical analyses.

### Statistical analyses

In this study, the student t-test and one-way analysis of variance were used to test the significance of means of continuous measurements among groups. Logarithmic transformation was performed for continuous random variables with positive skewness. The Chi-square test was used to test the significance of the associations between DM status and categorical variables. The effects of age, sex, and DM on the carotid hemodynamic parameters were assessed by the generalized linear regression analyses. The odds ratio (OR), which was estimated by the unconditional logistic regression model, was used as the indicator of the strength of association between carotid hemodynamic parameters and DM status. To assess the independent effects of carotid hemodynamic parameters on DM, we used multivariable logistic regression analyses to control for the confounding effects of other conventional cardiovascular risk factors. The area under the receiver operating curve (AUROC) was used as the indicator of the predictability of the regression model on DM. To explore whether there were interactions between hemodynamic biomarkers and other significant factors on the likelihoods of having DM, we carried out stratified analyses. For continuous variables, the values close to the medians in the non-DM subjects were used as the cut-points. We used the statistical method proposed by Clogg et al.^29^ to test the significance in the regression coefficients between two groups. All statistical analyses were performed using SAS 9.4 (SAS Institute Inc., Cary, NC, USA).

### Ethics approval and consent to participate

The study complied with the 1975 Helsinki Declaration on ethics in medical research and was reviewed and approved by the institutional review boards of MacKay Medical College (No. P990001, granted date: 2010/7/5) and MacKay Memorial Hospital (No. 14MMHIS075, Granted date: 2014/5/23).

## Results

Among 3892 participants, 417 (10.7%) of them fulfilled the DM definition and were regarded as cases. Table 1 shows that all baseline anthropometric and biochemical measurements, except for alcohol drinking, were significantly different between DM cases and non-DM controls. As compared to the non-DM controls, DM cases had significantly higher means of age, body mass index (BMI), waist circumference (WC), hip circumference (HIP), waist-to-hip ratio (WHR), blood pressure, and Log (TG) and higher proportions of the male sex, hypertension, schooling years < 12 years, and cigarette smoking. The means of TCHO, LDL-C, and HDL-C of DM cases were significantly lower than those of the non-DM controls.

**Table 1:** Comparisons of the baseline anthropometric and biochemical measurements between DM cases and non-DM controls.

Variables	Non-DM controls (n = 3475)		DM patients (n = 417)		*P*-value
Continuous variables	Mean	SD	Mean	SD	
Age (years)	55.6	8.9	60.0	8.3	< 0.0001
Body mass index (kg/m^2^)	24.4	3.5	26.2	3.8	< 0.0001
Waist circumference (cm)	85.0	10.0	90.6	9.9	< 0.0001
Hip circumference (cm)	96.2	7.1	98.2	7.7	< 0.0001
Waist-to-hip ratio (%)	88.2	7.1	92.3	6.6	< 0.0001
SBP (mmHg)	125.8	18.7	131.8	17.3	< 0.0001
DBP (mmHg)	76.1	12.6	78.3	12.1	0.0006
Total cholesterol (mg/dL)	205.9	37.7	195.7	43.8	< 0.0001
LDL (mg/dL)	122.2	32.1	114.5	35.1	< 0.0001
HDL (mg/dL)	56.5	15.0	48.8	12.5	< 0.0001
LDL-/HDL-C ratio	2.31	0.84	2.46	0.89	< 0.0001
Log_n_(TG)	4.57	0.55	4.87	0.58	< 0.0001
Categorical variables	n	%	n	%	
Female	2294	66.0	229	54.9	< 0.0001
Schooling years<12 years	2145	61.8	303	72.7	< 0.0001
Hypertension	734	21.0	188	45.1	< 0.0001
Cigarette smoking	704	20.3	123	29.6	< 0.0001
Alcohol drinking	443	12.8	55	13.2	0.81

Multivariable logistic regression analyses of the conventional cardiovascular risk factors showed that older age, hypertension, fewer schooling years, cigarette smoking, higher BMI, higher WHR, and higher TG were correlated with significantly higher ORs of having DM (Table 2). The multivariable-adjusted ORs of having DM with TCHO and HDL-C levels were significantly inverse. The multivariable-adjusted ORs for per 1.0 SD increases in BMI, WHR, TCHO, HDL-C, and log(TG) were 1.24 (95% CI 1.11–1.40), 1.30 (95% CI 1.14–1.48), 0.77 (95% CI 0.67–0.87), 0.84 (95% CI 0.71–0.99), and 1.98 (95% CI 1.56–2.51) respectively.

**Table 2:** Association analyses for DM with baseline clinical characteristics.

Variable	Age-sex adjusted		Multi-variable	
	OR^a^	(95% CI)	OR^b^	(95% CI)
Age (per 10 years)	1.33^**^	(1.25–1.41)	1.24^***^	(1.16–1.33)
Male sex	1.52^**^	(1.24–1.88)	–	
Schooling years<12 years (Y/N)	1.52^**^	(1.20–1.92)	1.42^*^	(1.11–1.81)
Hypertension (Y/N)	2.39^***^	(1.91–2.98)	1.55^**^	(1.23–1.97)
Cigarette smoking (Y/N)	1.63^**^	(1.24–2.13)	1.36^*^	(1.02–1.82)
Body mass index (per 1.0 SD)	1.60^***^	(1.45–1.77)	1.24^**^	(1.11–1.40)
Waist circumference (per 1.0 SD)	1.62^***^	(1.45–1.81)	–	
Hip circumference (per 1.0 SD)	1.26^***^	(1.14–1.39)	–	
Waist-to-hip ratio (per 1.0 SD)	1.65^***^	(1.47–1.86)	1.30^***^	(1.14–1.48)
SBP (per 1.0 SD)	1.25^***^	(1.13–1.39)	–	
DBP (per 1.0 SD)	1.16^**^	(1.05–1.28)	–	
Total cholesterol (per 1.0 SD)	0.78^***^	(0.70–0.87)	0.77^***^	(0.67–0.87)
LDL-C (per 1.0 SD)	0.79^***^	(0.71–0.88)	–	
HDL-C (per 1.0 SD)	0.54^***^	(0.47–0.62)	0.84^**^	(0.71–0.99)
LDL-/HDL-C ratio (per 1.0 SD)	1.16^**^	(1.05–1.29)	–	
Log(TG) (per 1.0)	2.52^***^	(2.09–3.04)	1.98^***^	(1.56–2.51)

–, not included; ^*^, 0.005<*p*<0.05; ^**^, 0.0001<*p*<0.005; ^***^, *p*<0.0001.

^a^ORs for age and sex were obtained from the model containing age and sex. ORs for other variables were adjusted for age and sex separately.

^b^ORs were obtained from the same best-fit regression model.

*CI* confidence interval, *OR* odds ratio, *SD* standard deviation.

The effects of age, sex, and DM on carotid blood flows, RI, and PI are shown in Table 3. As compared to female subjects, male subjects had significantly higher means of PSV, PI, and RI and significantly lower means of EDV and MFV (all *p*-values < 0.0001). The means of these five carotid hemodynamic parameters were all significantly different among seven age groups (all *p*-values < 0.0001). The means (SD) of PSV, EDV, and MFV for subjects aged 40–44 years were 95.1 (17.6) cm/sec, 26.1 (5.6) cm/sec, and 44.4 (8.0) cm/sec, respectively, for subjects aged 55–59 years were 84.5 (17.4) cm/sec, 24.4 (5.4) cm/sec, and 41.4 (7.9) cm/sec, respectively, and for subjects aged 70–74 years were 75.9 (16.7) cm/sec, 18.2 (4.6) cm/sec, and 34.5 (7.2) cm/sec, respectively. The means of RI and PI were lower for subjects aged 45–54 years and were higher for elderly subjects. Table 3 also shows that DM cases had significantly lower means of PSV, EDV, and MFV and significantly higher means of RI and PI as compared to the non-DM controls (all *p*-values < 0.0001).

**Table 3:** Effects of age, sex, and DM on PSV, EDV, MV, R1, and PI.

Variable	n	PSV (cm/sec)		n	EDV (cm/sec)		n	MFV (cm/sec)		n	RI		n	PI	
		Mean	(SD)		Mean	(SD)		Mean	(SD)		Mean	(SD)		Mean	(SD)
Total	3892	85.9	(18.3)		23.9	(5.9)		41.1	(8.5)		0.718	(0.052)		1.53	(0.33)
Sex															
Female	2523	84.6	(17.9)		24.8	(5.9)		42.3	(8.6)		0.705	(0.049)		1.43	(0.26)
Male	1369	88.3	(18.7)		22.4	(5.6)		38.9	(7.9)		0.743	(0.049)		1.72	(0.35)
*p*-value^1^		<0.0001			<0.0001			<0.0001			<0.0001			<0.0001	
Age (years)															
40–44	529	95.1	(17.6)		26.1	(5.6)		44.4	(8.0)		0.721	(0.054)		1.58	(0.35)
45–49	580	91.1	(18.7)		26.4	(5.6)		44.3	(8.6)		0.707	(0.049)		1.48	(0.29)
50–54	661	88.5	(18.2)		25.4	(5.9)		42.8	(8.5)		0.710	(0.052)		1.50	(0.35)
55–59	710	84.5	(17.4)		24.4	(5.4)		41.4	(7.9)		0.708	(0.050)		1.47	(0.29)
60–64	660	81.4	(16.3)		22.6	(5.3)		39.2	(7.7)		0.720	(0.050)		1.52	(0.31)
65–69	547	79.3	(16.4)		20.7	(4.7)		37.1	(7.1)		0.735	(0.050)		1.60	(0.33)
70–74	205	75.9	(16.7)		18.2	(4.6)		34.5	(7.2)		0.757	(0.046)		1.69	(0.32)
*p*-value^2^		<0.0001			<0.0001			<0.0001			<0.0001			<0.0001	
DM															
No	3475	86.5	(18.4)		24.3	(5.9)		41.6	(8.5)		0.716	(0.051)		1.52	(0.32)
Yes	417	80.9	(17.1)		21.1	(5.4)		37.5	(7.6)		0.736	(0.055)		1.62	(0.34)
*p*-value^1^		<0.0001			<0.0001			<0.0001			<0.0001			<0.0001	
Effect analyses^3^		b_1_	SE(b_1_)		b_1_	SE(b_1_)		b_1_	SE(b_1_)		b_1_	SE(b_1_)		b_1_	SE(b_1_)
Age (per 5.0 years)		− 3.17^**^	(0.16)		− 1.17^**^	(0.05)		− 1.51^**^	(0.07)		0.0038^**^	(0.0004)		0.0079^*^	(0.0027)
Sex (M/F)		4.38^**^	(0.58)		− 2.07^**^	(0.18)		− 3.06 ^**^	(0.26)		0.037^**^	(0.0016)		0.284^**^	(0.010)
DM (Yes/No)		− 3.28^*^	(0.91)		− 1.94^**^	(0.28)		− 2.38 ^**^	(0.41)		0.013^**^	(0.0025)		0.061^**^	(0.015)

^1^*p*-value of student’s t test.

^2^*p*-value of one-way analysis of variance.

^3^b1 and SE (b1) for each hemodynamic parameter was obtained from the same generalized linear model that contained sex, age (continuous), and DM.

*EDV* end diastolic velocity, *MFV* mean flow velocity, *PI* pulsatility index, *PSV* peak systolic velocity, *RI* resistance index, *SD* standard deviation, *SE* standard error; ^*^, 0.0001<*p*<0.01; ^**^, *p*<0.0001.

The results of generalized linear regression analyses were also shown in Table 3. The age trends for PSV, EDV, and MFV were significantly negative while for RI and PI were significantly positive. The adjusted regression coefficients of PSV, EDV, MFV, RI, and PI per 5.0 years increase in age at enrollment were − 3.17 cm/sec, − 1.17 cm/sec, − 1.51 cm/sec, 0.0038, and 0.0079, respectively (all *p*-values < 0.005). As compared to female subjects, male subjects had significantly higher adjusted means for PSV, RI, and PI, while exhibiting significantly lower adjusted means for EDV and MFV (all *p*-values < 0.0001). After adjustment for the effects of age and sex, the effects of DM status on all five carotid hemodynamic parameters remained statistically significant. As compared to the non-DM controls, the adjusted means of PSV, EDV, and MFV were − 3.28 cm/sec (*p *= 0.0003), − 1.94 cm/sec (*p *< 0.0001), and − 2.38 cm/sec (*p *< 0.0001), respectively, lower for the DM cases. The age-sex-adjusted means of RI and PI of DM cases were 0.013 and 0.0061 (both *p*-values < 0.0001), respectively, higher than those of the non-DM controls.

Table 4 shows that the prevalence rates of DM were negatively correlated with increased levels of PSV, EDV, and MFV and were positively correlated with increased levels of RI and PI. The prevalence rates of DM for subjects whose carotid blood flows were of the lowest quartile (Q1) and the highest quartile (Q4) ranged from 14.1 to 17.5% and from 4.9 to 5.1%, respectively. The prevalence rates of DM for subjects who had Q1 levels of RI or PI were approximately 7.0% and for Q4 levels of RI or PI were approximately 16.0%. As compared to subjects who had Q1 levels of carotid blood flows, subjects who had Q4 levels of PSV, EDV, and MFV had significantly decreased ORs of having DM. The corresponding age-sex-adjusted ORs were 0.51 (95% CI 0.37–0.72), 0.37 (95% CI 0.26–0.54), and 0.40 (95% CI 0.28–0.57), respectively. The age-sex-adjusted ORs were significantly increased for subjects who had Q3 and Q4 levels of RI and PI as compared to those who had Q1 levels of RI and PI.

**Table 4:** Association analyses for carotid blood flows, RI, and PI with DM.

	n	DM		Age-sex adjusted		Multivariable^1^	
		n	(%)	OR	(95% CI)	OR	(95% CI)
PSV (cm/sec)							
<72.78	969	137	(14.1)	1.00		1.00	
72.78–85.01	977	115	(11.8)	0.87	(0.66–1.14)	0.91	(0.68–1.21)
85.02–97.52	973	105	(10.8)	0.86	(0.65–1.14)	0.93	(0.69–1.26)
≥ 97.53	973	60	(6.2)	0.51^***^	(0.37–0.72)	0.59^**^	(0.41–0.83)
per 5 cm/sec increase				0.94^**^	(0.91–0.97)	0.95^**^	(0.92–0.98)
EDV (cm/sec)							
< 19.71	972	170	(17.5)	1.00		1.00	
19.71–23.71	974	122	(12.5)	0.82	(0.63–1.06)	0.89	(0.68–1.18)
23.72–27.81	973	77	(7.9)	0.55^***^	(0.40–0.73)	0.63^**^	(0.46–0.87)
≥ 27.82	973	48	(4.9)	0.37^***^	(0.26–0.54)	0.45^***^	(0.31–0.66)
per 5 cm/sec increase				0.69^***^	(0.62–0.76)	0.74^***^	(0.66–0.83)
MFV (cm/sec)							
< 35.13	973	163	(16.8)	1.00		1.00	
35.13–40.66	973	114	(11.7)	0.76^*^	(0.58–0.99)	0.81	(0.61–1.06)
40.67–46.84	973	90	(9.2)	0.67^*^	(0.50–0.89)	0.81	(0.60–1.09)
≥ 46.85	973	50	(5.1)	0.40^***^	(0.28–0.57)	0.53^***^	(0.37–0.77)
per 5 cm/sec increase				0.81^***^	(0.75–0.86)	0.86^***^	(0.80–0.93)
RI							
< 0.683	973	70	(7.2)	1.00		1.00	
0.683–0.720	973	77	(7.9)	1.00	(0.71–1.40)	0.93	(0.65–1.32)
0.721–0.752	973	112	(11.5)	1.50^*^	(1.09–2.07)	1.35^+^	(0.97–1.89)
≥ 0.753	973	158	(16.2)	1.84^*^	(1.33–2.53)	1.61^**^	(1.15–2.25)
per 0.1 increase				1.71^***^	(1.38–2.13)	1.52^**^	(1.21–1.91)
PI							
< 1.30	973	69	(7.1)	1.00		1.00	
1.30–1.48	973	87	(8.9)	1.19	(0.85–1.66)	1.09	(0.77–1.55)
1.49–1.70	973	108	(11.1)	1.46^*^	(1.05–2.03)	1.28	(0.91–1.80)
≥ 1.71	973	153	(15.7)	1.97^***^	(1.42–2.73)	1.58^**^	(1.12–2.23)
per 0.1 increase				1.88^**^	(1.36–2.60)	1.49^*^	(1.05–2.12)

^*^, 0.005<*p*<0.05; ^**^, 0.0001<*p*<0.005; ^***^, *p*<0.0001.

^1^Adjusted for age, sex, education, cigarette smoking, hypertension, body mass index, waist-to-hip ratio, and levels of total cholesterol and HDL-C.

*CI* confidence interval, *EDV* end-diastolic velocity, *MFV* mean flow velocity, *PI* pulsatility index, *OR* odds ratio, *PSV* peak systolic velocity, *RI* resistance index.

The results of multivariable analyses showed that the multivariable-adjusted ORs of having DM remained statistically significant for subjects who had Q4 levels of PSV, EDV, MFV, RI, and PI, relative to those with Q1 levels (Table 4). The corresponding multivariable-adjusted ORs of having DM were 0.59 (95% CI 0.41–0.83), 0.45 (95% CI 0.31–0.66), 0.53 (95% CI 0.37–0.77), 1.61 (95% CI 1.15–2.25), and 1.58 (95% CI 1.12–2.23), respectively. As compared to those who had a Q1 level of EDV, subjects who had a Q3 level of EDV also had a significantly lower OR (0.63; 95% CI 0.46–0.87). The multivariable-adjusted ORs of having DM per 5.0 cm/sec increase in PSV, EDV, and MFV were 0.95 (95% CI 0.92–0.98), 0.74 (95% CI 0.66–0.83), and 0.86 (95% CI 0.80–0.93), respectively. Increased PI and RI were significantly positively correlated with the likelihood of DM. The multivariable-adjusted ORs of having DM per 0.1 increases in RI was 1.52 (95% CI 1.21–1.91) and for per 1.0 increase in PI was 1.49 (95% CI 1.05–2.12).

The comparisons of the predictabilities of the regression models that contained different carotid hemodynamic parameters are shown in Table 5. The AUROC for the basic model, i.e., the most predictive model selected from the regression analyses which contained all significantly conventional cardiovascular risk factors, was 0.7578 (95% CI 0.7346–0.7809). The results of multivariable logistic regression analyses showed that EDV was the most significantly independent predictor of DM. The AUROC were 0.7658 (95% CI 0.7430–0.7885) and 0.7678 (95% CI 0.7453–0.7904) for models adding EDV as a continuous or a categorical variable, respectively. The additions of PSV and MFV also significantly increased the predictabilities of DM status but with smaller added AUROC (Table 5).

**Table 5:** Comparisons of the predictive abilities of models contained different markers of carotid ultrasonography.

	AUROC		Added AUROC	
	(%)	(95% CI)	(%)	(95% CI)
Basic model^1^	75.78	(73.46–78.09)	Ref.	
+ PSV (quartile)	76.20	(73.93–78.47)	0.43^*^	(0.00–0.89)
+ PSV (continuous)	76.18	(73.89–78.47)	0.41^*^	(0.01–0.81)
+ EDV (quartile)	76.58	(74.30–78.85)	0.80^*^	(0.15–1.46)
+ EDV (continuous)	76.78	(74.53–79.04)	1.00^*^	(0.29–1.73)
+ MFV (quartile)	76.29	(74.00–78.57)	0.51^*^	(0.26–1.02)
+ MFV (continuous)	76.39	(74.11–78.67)	0.61^*^	(0.08–1.15)
+ RI (quartile)	76.23	(73.93–78.54)	0.46^+^	(–0.08–1.00)
+ RI (continuous)	76.00	(73.67–78.33)	0.23	(–0.30–0.75)
+ PI (quartile)	75.92	(73.59–78.25)	0.14	(–0.27–0.52)
+ PI (continuous)	75.86	(73.54–78.19)	0.09	(–0.22–0.39)

^+^, 0.05<*p*<0.10; ^*^, *p*<0.05.

^1^Include age, sex, education, cigarette smoking, hypertension, body mass index, waist-to-hip ratio, and levels of total cholesterol and HDL-C.

To explore whether there were interactive effects between EDV and conventional CVD risk factors on the likelihoods of having DM, we carried out stratified analyses. Table 6 shows that increased EDV were correlated with significantly decreased ORs of having DM in all strata. The regression coefficient (SE) for per 5 cm/sec increase in EDV for subjects aged < 55 year was non-significantly different that of subjects aged ≥ 55 years (− 0.229 (0.096) vs. − 0.414 (0.069), *p *= 0.12). Similarly, there was no significant difference in the regression coefficients between two strata of other factors.

**Table 6:** Stratified analyses for having DM with EDV.

	Multivariable logistic regression model^1^				
	b_1_	(SE)	*p*^2^	OR	(95% CI)
Age (years)					
< 55	− 0.229^*^	(0.096)	0.12	0.80	(0.66–0.96)
≥ 55	− 0.414^**^	(0.069)		0.66	(0.58–0.76)
SEX					
Male	− 0.323^*^	(0.086)	0.71	0.72	(0.61–0.86)
Female	− 0.279^*^	(0.077)		0.76	(0.65–0.88)
Schooling years < 12 years					
Yes	− 0.291^**^	(0.068)	0.77	0.75	(0.66–0.85)
No	− 0.328^*^	(0.106)		0.72	(0.59–0.89)
Cigarette smoking					
No	− 0.272^**^	(0.067)	0.42	0.76	(0.67–0.87)
Yes	− 0.374^*^	(0.108)		0.69	(0.56–0.85)
Hypertension					
No	− 0.303^**^	(0.074)	0.74	0.74	(0.64–0.85)
Yes	− 0.343^*^	(0.092)		0.71	(0.59–0.85)
Body mass index					
< 25 Kg/m^2^	− 0.401^**^	(0.088)	0.24	0.67	(0.56–0.80)
≥ 25 Kg/m^2^	− 0.266^*^	(0.075)		0.77	(0.66–0.89)
Waist-to-hip ratio					
< 90%	− 0.343^*^	(0.090)	0.54	0.71	(0.59–0.85)
≥ 90%	− 0.272^*^	(0.074)		0.76	(0.66–0.88)
Total cholesterol					
< 200 mg/dL	− 0.326^**^	(0.079)	0.65	0.72	(0.62–0.84)
≥ 200 mg/dL	− 0.275^*^	(0.083)		0.76	(0.65–0.89)
HDL-C					
< 50 mg/dL	− 0.344^**^	(0.079)	0.47	0.71	(0.61–0.83)
≥ 50 mg/dL	− 0.262^*^	(0.083)		0.77	(0.66–0.91)
Log_n_(TG)					
< 4.6	− 0.430^**^	(0.097)	0.11	0.65	(0.54–0.79)
≥ 4.6	− 0.239^*^	(0.070)		0.79	(0.69–0.90)

^1^per 5 cm/sec increase in EDV and adjusted for all, except for the stratified variable, significant factors (including age, sex, education, cigarette smoking, hypertension, body mass index, waist-to-hip ratio, and levels of total cholesterol and HDL-C).

^2^*p*-value for the comparison of regression coefficients between two groups.

*CI* confidence interval, *EDV* end-diastolic velocity, *OR* odds ratio, *SE* standard error; ^*^, 0.0001<*p*<0.01; ^**^, *p*<0.0001.

## Discussion

In this study, we conducted a community-based case-control study, in which we enrolled approximately 4000 subjects aged 40–74 residing in the northern coastal area of Taiwan. In the case-control study, large numbers of DM cases and non-DM controls received color Doppler ultrasonographic measurements, including PSV, EDV, MFV, PI, and RI. We found significant age and sex effects on these hemodynamic parameters. After adjustment for the effects of age and sex, all these five carotid hemodynamic parameters remained significantly influenced by DM status. As compared to the non-DM controls, the adjusted means of PSV, EDV, and MFV were significantly lower and the adjusted means of RI and PI were significantly higher for the DM cases. We also found that after controlling for the effects of other conventional CVD risk factors, the multivariable-adjusted ORs of having DM were negatively correlated with PSV, EDV, and MFV and were positively correlated with PI and RI. More importantly, the additions of PSV, EDV, and MFV, either in categorical or continuous scales, significantly improved the predictabilities of the regression models on DM status and among them EDV was the most significantly independent predictor.

Pulsatility is a crucial aspect of the cardiovascular system, linked to artery elasticity. The natural pressure pulsations from each left ventricle contraction are reduced by the elasticity of large arteries. The aorta's expansion stores part of the stroke volume, lessening pulsatile stress on microvasculature^30^. However, with the loss of elastic fiber with age and disorders of metabolism, such as hyperlipidemia or DM, arterial walls continually increase their stiffness resulting in a gradual increase in blood pressure and, eventually affecting global cardiovascular health^16^. Pulsatile hemodynamics can be measured with invasive or non-invasive methods. Inserting an intraarterial catheter is the most accurate method of assessing pulsatile hemodynamics, however, multiple studies indicated that non-invasive methods could be reasonable surrogates for invasive ones^31–33^. Hemodynamic parameters including blood velocities such as PSV, EDV, and MFV as well as PI and RI were used to study their clinical correlation with different cardiovascular conditions including but not limited to stroke^34–39^, hypertension^40–42^, arterial stenosis^43–46^.

Prolonged hyperglycemia in patients with DM can damage the vascular endothelium leading to an increase in vascular stiffness and likely a change in hemodynamics^47^. The increase in the stiffness of large vessels can result in increased pulsation and microvascular complications^48^. Several studies have shown possible applications of hemodynamic parameters in predicting and preventing microvascular complications. In 2000, Lee et al. first studied 56 type 2 DM patients and 70 controls and measured their flow velocities and PI of the middle cerebral artery (MCA), extracranial internal carotid artery (ICA), and basilar artery (BA)^25^. They found that PIs of the MCA and ICA were closely correlated with the duration of DM. Some of these studies were lack of sex and age-matched controls^49–52^ while some studies were designed to test the effect of drugs with only DM patients^53,54^. In studies with sex and age-matched controls, Agha et al. measured the velocity and PI of BA, ICA, and MCA in 141 DM patients and 132 controls^55^; Dikanovic et al. measured the velocity and PI of MCA in 100 type 2 DM patients and 100 controls^26^; Park et al. measured the velocity and PI of MCA in 90 type 2 DM patients and 45 controls^56^; Zou et al. measured the velocity, PI and RI of dorsalis pedis artery and plantar digital artery in 56 type 2 DM patients and 50 controls^57^. All of these studies came to the same conclusion as we did that hemodynamic parameters including velocities, PI, and RI can be useful indicators and predictors of DM. However, none of them perform their studies at the same large scale as we did.

In a previous study, we included 4073 participants from the same study area, with prevalence rates of carotid plaque and DM at 35.4% and 11.3%, respectively^22^. The study found statistically significant linear trends between the likelihood of having DM and the total number of carotid plaques, maximum carotid stenosis, or severity of carotid atherosclerosis. The multivariate-adjusted odds ratio (OR) for DM was 1.57 (1.25–1.98), indicating a significantly higher risk for subjects with carotid plaques compared to those without observable plaque images. Furthermore, a greater number of carotid plaques, increased maximum carotid atherosclerosis, and more severe carotid atherosclerosis were associated with significantly higher ORs for DM. The prevalence rate of carotid plaque in the prevalent DM group was also significantly higher than in the incident DM group. In our most recent case-control study, we enrolled 309 carotid plaque-positive subjects and 439 carotid plaque-negative subjects from a community-based cohort^23^. Multivariable analyses of anthropometric attributes and biochemical profiles revealed that DM was a significant independent predictor in the best-fit regression model for the presence of carotid plaque. Among the 43 tested DM SNPs, 9 showed promising associations with carotid atherosclerosis, controlling for age, cigarette smoking, and hypertension. Although not all of these promising SNPs demonstrated significant independent effects in the multivariable analyses, a notable linear trend between their composite indicator 9-GCS and the risks of carotid atherosclerosis was observed. We identified four SNPs (rs9937354, rs10842993, rs7180016, and rs4383154) that exhibited significant independent effects with carotid atherosclerosis. Genes that are closely associated with these SNPs include FTO, PRC1, GP2, and KLHL42.

Several potential mechanisms of increased arterial stiffness and altered hemodynamics in DM have been implicated including the formation of advanced glycation endproducts (AGEs) and the dysregulation of nitric oxide (NO)^58^. The formation of AGE involves multiple reversible and irreversible steps, ultimately leading to the pathological binding of collagen molecules within the arterial vessel wall^59^. Numerous studies have linked AGEs to the acceleration of age-related vascular changes and the development of cardiovascular events in both diabetic and non-diabetic populations^60^. The presence of AGE-induced cross-links can make collagen highly resistant to enzymatic breakdown, resulting in a reduced degradation rate. This, in turn, contributes to the increased collagen content observed in arterial walls, which is a characteristic of aging and is further accelerated in conditions such as DM^61^. Research has shown a positive correlation between carotid-femoral pulse wave velocity and collagen crosslinking^62^. Moreover, the levels of specific AGEs in aortic tissue have been found to correlate with aortic stiffness in individuals with and without DM^63^. NO possesses various beneficial properties, including vasodilation, anti-platelet activity, anti-inflammatory effects, and antioxidant properties^64^. However, in the state of insulin resistance, the activation of NO synthase is impaired, and there is an increase in the production of superoxide. These factors together contribute to a decrease in the availability of NO^65^. In individuals with diabetes, particularly those with microvascular disease, basal levels of NO are reduced compared to those without such complications. Furthermore, the severity of microvascular disease correlates with a further decline in NO levels^66^. Further mechanical studies including gene-association studies based on our current findings will provide insight into finding therapeutic targets for atherosclerosis and related complications in DM patients.

## Conclusion

The findings of this study highlight a noteworthy association between DM and changes in hemodynamic parameters. Adding hemodynamic parameters enhanced the predictabilities of the regression models on DM status. Gaining a deeper understanding of this relationship can aid in identifying individuals who are at a heightened risk of DM. Future follow-up and mechanical studies will enlighten us on factors that contribute to the development of vascular complications in DM patients.

## Data Availability

The datasets used and/or analyzed during the current study are available from the corresponding author upon reasonable request.

## Abbreviations

AUROC: Area under the receiver operating curve
BMI: Body mass index
CCA: Common carotid artery
CHD: Coronary heart disease
TCHO: Total cholesterol
CI: Confidence interval
DBP: Diastolic blood pressure
DM: Diabetes mellitus
EDV: End-diastolic velocity
FPG: Fasting plasma glucose
FTG: Fasting triglycerides
HDL-C: High-density lipoprotein cholesterol
HIP: Hip circumference
LDL-C: Low-density lipoprotein cholesterol
MFV: Mean blood flow velocity
OR: Odds ratio
PI: Pulsatility index
PSV: Peak systolic velocity
RI: Resistance index
SBP: Systolic blood pressure
SD: Standard deviation
SE: Standard error
WC: Waist circumference
WHR: Waist-to-hip ratio
